# Optimization of Mushroom (*Agaricus bisporus* and *Pleurotus ostreatus*) By-Products Processing for Prospective Functional Flour Development

**DOI:** 10.3390/foods13244046

**Published:** 2024-12-14

**Authors:** Patricia Bermúdez-Gómez, Juana Fernández-López, Margarita Pérez-Clavijo, Manuel Viuda-Martos

**Affiliations:** 1 Mushroom Technological Research Center of La Rioja (CTICH), Carretera Calahorra, km 4, 26560 La Rioja, Spain; patricia.bermudez@goumh.umh.es (P.B.-G.); direccion@ctich.com (M.P.-C.); 2 IPOA Research Group, Institute for Agri-Food and Agri-Environmental Research and Innovation (CIAGRO-UMH), Miguel Hernández University, 03312 Alicante, Spain; j.fernandez@umh.es

**Keywords:** *Agaricus bisporus*, *Pleurotus ostreatus*, stem, by-products, bioeconomy, drying

## Abstract

Stems are a major by-product of mushroom production. This study optimizes the transformation of *Agaricus bisporus* stems (ABS) and *Pleurotus ostreatus* stems (POS) into flour. ABS are attached to the peat, so, the process was divided into two steps. First, four cleaning methods were tested for ABS: pre-drying, brushing, abrasive peeling, and immersion in chlorinated water and brushing. Abrasive peeling was the most effective, showing the lowest color difference (9.18), total aerobic count (3.48 log cfu/g), and the highest profitability (1 kg/h). In the second step, ABS and POS were dried using a freeze-dryer, a dehydrator, and an oven. Dehydration resulted in stems with a lower browning index (24.57 in ABS and 29.95 in POS) than the oven, and a smaller energy consumption than the freeze-dryer. Finally, three dehydration temperatures (40, 50, and 60 °C) were compared. Dehydration at 50 °C resulted in faster moisture loss (24 h) and similar phenolic compound concentrations (3.35 and 7.69 mg GAE/100g fresh ABS and POS, respectively) than at 40 °C (32 h in ABS and 28 h in POS). In conclusion, the transformation of ABS and POS into flours requires processes that influence their chemical composition, physicochemical characteristics, and the profitability of their production. In this project, the optimal process involved cleaning ABS through abrasive peeling and dehydrating both ABS and POS at 50 °C for 24 h.

## 1. Introduction

Edible mushrooms are widely consumed across the globe due to their appealing sensory qualities and exceptional nutritional profile. They are recognized for being low in calories and fat while providing significant amounts of protein, dietary fiber, and vitamins [1,2,3,4]. Moreover, studies in recent decades have demonstrated their potential biological properties, including anti-inflammatory, anticancer, antihyperglycemic, antibacterial, and antioxidant effects [5,6,7,8,9]. These health benefits are linked to the presence of secondary metabolites [7].

The exceptional sensory qualities and health benefits of fruiting bodies have fueled the global cultivation of edible mushrooms since the late 1990s. In general, mushroom production worldwide has shown consistent growth since 1999, reaching a value of 46,608,283 thousand of USD in 2021 [4,10]. The most widely known varieties include *Agaricus bisporus*, *Lentinula edodes*, and *Pleurotus* species [11]. Specifically, *A. bisporus* (AB), is among the most extensively cultivated edible mushrooms globally, representing 54% of the total production of edible fungi [5,12]. The second most cultivated species is the genus *Pleurotus*, with *Pleurotus ostreatus* (PO) being the most significant species [13,14]. The global production of this genus accounts for approximately 27% of the total edible fungi produced [5]. Throughout the commercialization of fruiting bodies, substantial quantities of by-products are produced, arising from two main processes: mushroom cultivation and harvesting. During cultivation, the primary waste material generated is spent mushroom substrate, also known as mushroom compost. In the harvesting phase, the discarded materials include mushroom stems and those mushrooms that do not meet the required size or shape for sale. Stems or stipes can constitute up to 20% of the total production yield [15,16,17]. This waste is typically handled through composting or incineration, both of which pose environmental concerns due to the release of unpleasant odors, the attraction of disease-carrying insects, and air pollution [12,18,19]. To achieve the goals outlined in the 2030 Agenda for Sustainable Development and considering the economic aspects of the food industry, it is essential to find new, alternative, and profitable solutions for valorizing agricultural waste. Depending on the properties of the various by-products, they could be applied in the food, energy, pharmaceutical, and cosmetic sectors [20]. *A. bisporus* stems (ABS) and *P. ostreatus* stems (POS) contain numerous bioactive compounds such as polysaccharides (β-D-glucans and chitin), minerals (zinc, iron, manganese, etc.), vitamin precursors (ergosterol), and amino acids (glutamic acid, alanine, valine, isoleucine, etc.) [1,3,4,16,21,22]. In recent years, the potential of this by-product has been explored as a source for extracting valuable compounds such as ergosterol, peptides, phenolic compounds, glucans, and chitin [1,12,17,18,23,24]. 

Although numerous ‘green’ extraction technologies have been developed in recent years, such as pressurized liquids or supercritical fluids, these processes are expensive, still use cosolvents, and generate additional waste. While these technologies focus on the extraction of valuable compounds from agro-industrial co-products, an alternative approach to revalorize these stems could be their incorporation directly into food products. Given sustainability goals and the economics of the food industry, transforming co-products into flour offers a cost-effective and environmentally friendly option, turning the by-product into an upcycled ingredient for the food industry. As far as our knowledge, this methodology has been proven in noodles and vegan nuggets, among others [14,25]. However, the procedure for cleaning and processing these products for incorporation into food has never been extensively described. In order to incorporate co-products into food, strict traceability, safety, and hygiene measures must be met. The food industry co-products generally have high moisture content and water activity, which could lead to microbiological growth and lower self-life. 

In this context, the main objective was to valorize the co-products (steams) from the commercialization of edible mushrooms (*A. bisporus* and *P. ostreatus*) obtaining a stable intermediate ingredient (flour type) by applying only physical processes, including cleaning and drying to keep food safety. Additionally, different drying methods (equipment, temperature, and time) were evaluated to identify the more feasible option for large-scale implementation in the food industry

## 2. Methodology

### 2.1. Fungi Processing

**Figure 1:**
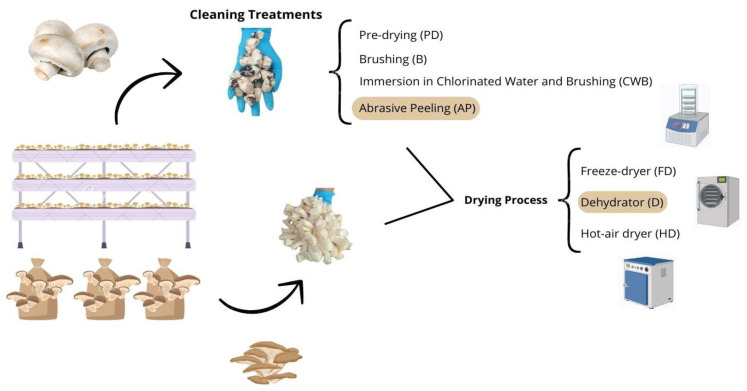
Fungi processing scheme.

A scheme of the fungi processing is shown in Figure 1. The *Agaricus bisporus* stems, from the Sylvan A15 strain, were provided by Cultivos Riojal (Autol, La Rioja, Spain) and were freshly cut after the fruiting bodies were collected. *A. bisporus* was cultivated on a substrate primarily composed of wheat straw, chicken manure, and recirculated process water, with temperatures maintained between 15 and 19 °C and relative humidity ranging from 87 to 91% over a growth period of 8 days. The samples were stored at 4 °C for no longer than 5 days before the cleaning treatments were applied. The *Pleurotus ostreatus* stems from the Sylvan SPOPPO strain were supplied by Micotec SA (Autol, La Rioja, España) and were freshly cut after the fruiting bodies were collected. *P. ostreatus* was cultivated on a substrate primarily composed of wheat straw, with temperatures ranging from 18 to 23 °C and relative humidity between 90 and 95% over a growth period of 10 days. The samples were kept at 4 °C for no more than 5 days until the drying process.

#### 2.1.1. ABS Cleaning Treatments

Peat is used as a layer on the growing substrate of AB to maintain moisture and encourage mushroom fruiting body production [26]. This peat is adhered to the stems, which means ABS samples must be cleaned before drying. However, POS grows on wheat straw substrate, and during the subsequent drying process, the straw detaches naturally. As a result, there is no need for the same pre-treatment cleaning step for POS, unlike ABS. For the cleaning optimization of ABS, samples were separated into five analysis groups as follows:

- Control fresh (CF)

**Figure 2:**
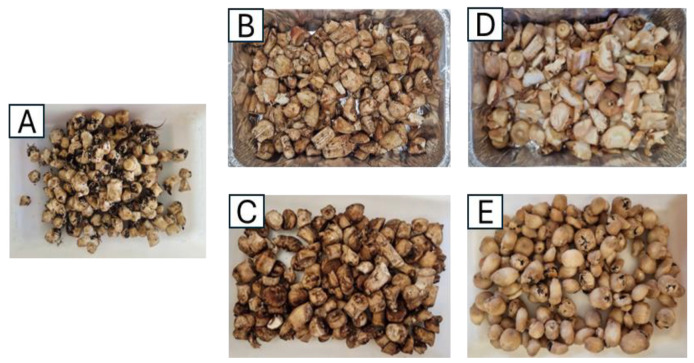
ABS cleaning treatments. (**A**)—control fresh, (**B**)—pre-drying, (**C**)—brushing, (**D**)—immersion in chlorinated water and brushing, (**E**)—abrasive peeling.

ABS were received after fruiting body harvesting. They were then stored at 4 °C for no more than 5 days before being dried. Figure 2 shows the ABS control fresh group without cleaning treatment applied.

- Pre-drying (PD)

ABS were dried in a dehydrator (Master Jerky 550, Klarstein, Chal-Tec GmbH, Berlin, Germany) for 3 h at 40 °C to dry peat and separate it easier from the stem. Later, peat was removed by erosion with a steel mesh with a 0.23 mm mesh light. The result is shown in Figure 2.

- Brushing (B)

ABS were brushed one by one with a wet nylon bristle brush (34 × 10 cm) of medium stiffness. Figure 2 shows the result of this cleaning process. It is important to note that the entire brushing process was carried out by a single person throughout the experiment, which eliminated any variability related to the skills or techniques of different operators. To ensure consistency, the same person used the same tools and followed the same standardized protocol throughout the study. This approach helped minimize the impact of interpersonal differences on the results of the cleaning process.

- Immersion in chlorinated water and brushing (CWB)

Chlorinated water was prepared by adding sodium hypochlorite solution to tap water to obtain concentrations of 100 mg/L free chlorine. Initially, ABS were immersed in chlorinated water for 10 min. This process disinfected the stems and softened the peat so it could be removed more easily with the nylon bristle brush specified in the previous section. The result is shown in Figure 2.

- Abrasive peeling (AP)

ABS were cleaned in this method with pressurized water and an abrasive surface. This method was carried out in an automated peeling system (PP-12, Sammic, Serhs Equipment, Madrid, Spain). The automated peeling has a capacity of 30 kg and a rotating disk with sanding material that allows for the peat to be removed together with pressurized water. The abrasion was applied for 30 s for each 0.5 kg of ABS; these conditions were selected for this experiment based on previous studies carried out by the research group, in which the physical structure of the mushroom co-products were considered as well as energy and water consumption. Figure 2 shows the result. 

All samples were processed for the rest of the analysis by dehydrated in a dehydrator (Master Jerky 550, Klarstein, Chal-Tec GmbH, Berlin, Germany) for 24 h at 40 °C and grounded in ultra-centrifugal mills (ZM 200, Retsch™, Thermo Fhisher Scienctific, Düsseldorf, Germany) at a sample size of 1 mm. In concordance with the results of color, protein, moisture, yield, total phenolic content, total aerobic count, and profitability, one cleaning method was selected to obtain the ABS that would be used in the drying optimization.

#### 2.1.2. Drying Process 

ABS and POS were dried with three different equipment. The conditions were selected based on previous studies carried out by the research group, in which the thermal stability, moisture content, and physical structure of the mushroom co-products were considered. Powder moisture was below 12% to ensure the stability of the samples [20]:

- Freeze-dryer (FD)

ABS and POS were cut into pieces of no more than 5 square cm, frozen at −80 °C, and freeze-dried in a freeze-dryer (Alpha 1–2 LD plus, Christ, Sigma Laborzentrifugen GmbH, Osterode am Harz, Germany) with a capacity of 28 kg. The process was carried out at a chamber pressure of 0.200 atm, a cold collector temperature of −54 °C, and a chamber temperature of 40 °C for 24 h.

- Dehydrator (D)

ABS and POS were cut into 5 mm thick slices and dried in a dehydrator with a capacity of 36 kg (Master Jerky 550, Klarstein, Chal-Tec GmbH, Berlin, Germany) for 24 h at 40 °C.

- Hot-air dryer (HD)

ABS and POS were cut into 5 mm thick slices and dried in a universal oven with a capacity of 45 kg (UFB 400, Memmert, I.C.T., S.L., La Rioja, Spain) for 24 h at 40 °C with a ventilation power of 100%.

One drying method was selected based on color and energy consumption. Subsequently, the drying conditions were optimized for the selected method by drying ABS and POS at 40 °C, 50 °C, and 60 °C until the moisture content was less than 5%. POS was cut into 5 mm thick slices before being dried. ABS was not cut into slices in order to eliminate another processing factor that could potentially cause physicochemical changes, as well as to avoid increasing the cost of the final product. Dehydrated samples were processed in ultra-centrifugal mills (model ZM 200, Retsch™, Düsseldorf, Germany) to achieve a sample size of 1 mm, resulting in the flours.

### 2.2. Moisture Content (MC)

The moisture content was determined by following the AOAC 934.06, with some modifications [27]. All measurements were conducted in triplicate and reported as g/100 g of fresh. The moisture content was calculated by measuring the weight difference before and after drying at 55 °C in an ED 400 stove (Binder, Tuttlingen, Germany) until a constant weight was achieved. The moisture level was recorded every 2 h throughout the drying process at 40, 50, and 60 °C to generate the drying curves for both ABS and POS.

### 2.3. Yield and Profitability

All determinations were performed in triplicate and expressed as g dw/100 g of fresh stem. Yield was calculated gravimetrically by the difference in weight before and after cleaning treatment and drying. All determinations were performed in triplicate and expressed as g dw/100 g of fresh. Profitability was calculated by dividing the yield by the time needed to process 1 g of fresh. The results were expressed as g dw/h.

### 2.4. Color

The color was measured in all samples of cleaning and drying optimization with a BGD 551 colorimeter (QL Instruments, La Rioja) with illuminant D65, observer 8°, SCI mode, 8 mm aperture for illumination, and 4 mm for measurement, based on the CIELab color space. The following color coordinates were determined: lightness (L*), redness (a* ± red-green), and yellowness (b* ± yellow-blue). From these coordinates, chroma (C*) was calculated using Equation (1). Color difference (ΔE*) was calculated based on the control fresh and freeze-dryer using Equation (2) and the browning index (BI) following Equation (3) [28].

(1) $$C^{*}=\sqrt{\left(a^{*2}+b^{*2}\right)}$$

(2) $${\Delta E}^{*}=\sqrt{{{(∆L}^{*})}^{2}+{{(∆a}^{*})}^{2}+{{(∆b}^{*})}^{2}}$$

(3) BI=100(x−0.31)/0.175

where $x={(a}^{*}+1.75L^{*})/\left(5.65L^{*}+a^{*}-3.01b^{*}\right)$

### 2.5. Quantification of Proteins Using a Bradford Assay 

Crude protein was determined in all samples of cleaning and drying optimization by following the Bradford method [29]. In total, 50 mg of each flour were combined with 1 mL of ultrapure water and vortexed using a RSLAB-6 PRO vortex (RSLab, Parana, Argentina) at 20,000 rpm for 1 min, and then were stirred at room temperature for 1 h. After centrifugation (5000× *g* for 10 min at 4 °C), 10 μL of the supernatant was mixed with 290 μL of Bradford reagent in a 96-well plate to conduct the assay. Bovine serum albumin (BSA) was prepared with distilled water and used as standard. Standard solutions for the standard curve were prepared (0.0–1.0 mg/mL BSA), and then 10 μL of all standards solutions were mixed with 290 μL of Bradford reagent. The absorbance was measured using a spectrophotometer (ThermoScientific, Madrid, Spain) at 595 nm. The sample was diluted if its absorbance at 595 nm was more than 2. The results were expressed as g protein/100 g of fresh. 

### 2.6. Assessment of the Total Phenolic Content

The procedure outlined by Delgado-Ospina et al. [30] was followed, with some modifications, to extract polyphenols and antioxidant compounds from ABS and POS. Briefly, 3.0 g of each sample were separately combined with 10 mL of a methanol:water mixture (80:20, *v*/*v*), vortexed in an RSLAB-6 PRO vortex (RSLab, Parana, Argentina) at 20,000 rpm for 1 min, and then subjected to sonication in an Ultrasons-HD (Selecta JP, Barcelona, Spain) for 10 min at 35 °C. After centrifugation (5000× *g* for 10 min at 4 °C), the supernatants were collected. The procedure was repeated twice with the remaining pellet. Lastly, the pellet was combined with 10 mL of acetone:water mixture (70:30, *v*/*v*), and the same steps were repeated. The supernatants were combined and evaporated to dryness using a SyncorePlus instrument (Büchi Labortechnik AG, Flawil, Switzerland). The dried extracts of ABS and POS were then re-dissolved in a methanol:water solution (80:20, *v*/*v*) prior to the subsequent analysis. Next, the total phenolic content (TPC) was measured using the Folin–Ciocalteu method as described by Liu et al. [31]. The results were reported as milligrams of gallic acid equivalents per 100 g of fresh (mg GAE/100 g of fresh product).

### 2.7. Total Aerobic Count (TAC)

In total, 10 g of each sample was taken aseptically, transferred to sterile plastic pouches, and homogenized for 1 min at room temperature with 90 mL of sterile peptone water using a homogenizer Smasher^TM^ (AES Blue Line^TM^, Biomerieux, Marcy-l’Étoile, France). After making serial dilutions, 1 mL of the 1:100,000 dilution sample was deposited onto a 3M Petrifilm Aerobic Count Plate (3M Health Care, Madrid, Spain) and incubated at 37 °C for 48 h. Each sample was conducted in triplicate, and TAC was expressed as log_10_ numbers of colony-forming units/gram (log cfu/g).

### 2.8. Statistical Analysis

For each experiment, three separate samples were analyzed, with three replications per sample. Data obtained for all the determinations were analyzed using one-way and two-way ANOVAs. Tukey’s post hoc test was applied for comparisons of means; differences were considered significant at *p* < 0.05. 

## 3. Results and Discussion

### 3.1. ABS Cleaning Treatments

#### 3.1.1. Effect of Cleaning Treatment on ABS Physicochemical Characteristics

The moisture content, yield, protein content, and total phenolic content of the stems were discretely influenced by cleaning treatment (*p* < 0.05). According to the results (Table 1), the water content in CF was within the range (81.8–94.8%) reported in a previous review article on edible mushrooms [32]. However, it was lower than the 91.5–92.1% described by Cherno et al. [33] in *A. bisporus* and *P. ostreatus* stems. This disagreement could be due to other parameters related to harvest, growth, and storage conditions [32]. Due to water use during both processes, the highest MC values (*p* < 0.05) are shown in CWB (88 g/100 g of fresh) and AP (87 g/100 g of fresh) treatments. Overall, the application of all treatments reduced the powder yield compared with the CF (*p* < 0.05). The lowest value (*p* < 0.05) of yield was shown in AP (7.29 g/100 g fresh). Even so, the revalue of the stems by applying only physical processes results in higher yields than other processes such as polysaccharides (4.7 g/100 g dw) and chitin extraction (27 g/100 g dw) [34,35]. 

All treatments reduced the protein content of the stems compared to the CF (*p* < 0.05). The protein content in CF (0.96 g/100 g fresh) was similar to the values previously reported in *A. bisporus* stems (1.23 g/100 fw) [36]. AP showed the lowest (*p* < 0.05) protein content (0.26 g/100g fresh). This could be attributed to the loss of peat, which contains 0.5 g/kg of nitrogen [37]. Regarding the total phenolic content, it is worth mentioning that CWB showed the highest value (*p* < 0.05). The effect of chlorinated water on the activity of enzymes related to phenolic compound decomposition and synthesis has been studied in previous works. This cleaning treatment showed a slight decrease in polyphenol oxidase (PPO) and peroxidase (POD) activity and enhanced phenylalanine ammonia-lyase (PAL) activity [38]. On the other hand, AP showed the lowest (*p* < 0.05) TPC. The use of the abrasive could enhance PPO and POD activity in the stems by disrupting tissue, thereby increasing surface contact with oxygen, while phenols would migrate into the intracellular space, promoting enzymatic catalysis [39]. PPO and POD activity are related to the browning index due to the formation of brown-pigmented melanin compounds as secondary metabolites [40]. However, this parameter (Table 2) was not the highest at this treatment, so in this case, phenolic compounds could have been lost by a combination of enzymatic activity and dissolving with the added water [39]. Moreover, the oxidative reaction could explain the lower TPC (*p* < 0.05) in PD and B compared with the CF.

The color parameters for the dehydrated stems in different cleaning treatments are shown in Table 2. Only B, CWB, and AP treatments displayed a significant effect (*p* < 0.05) on luminosity compared with CF. The lightness values (L*) of the samples with CWB and AP treatment were higher (*p* < 0.05) than the CF, and are thus less dark. Additionally, there was no difference between CWB and B (*p* < 0.05). 

The rise in L* with the cleaning treatment could be due to peat loss, which has a L* within the range of 12–35 [41]. PD showed the highest yellowness (b*), redness (a*), browning index (BI), and chroma (C*) values (*p* < 0.05), which could be due to the pre-drying step in this cleaning method. A higher b* in mushrooms is considered an indicator of color degradation, due to their temperature sensitivity [42]. Furthermore, a* and b* increases are related to hot air drying in previous works due to more oxidative and enzymatic reactions [43]. The parameters of a*, b*, C*, and BI values in B were also higher than CWB and AP due to a higher oxidative and enzymatic reaction due to tissue damage during brushing. It is worth noting that AP showed the smallest color difference ∆E*, which could be due to the lowest color degradation observed in the a*, b*, and BI parameters. 

#### 3.1.2. Effect of Cleaning Treatment on Total Aerobic Count and Profitability

**Figure 3:**
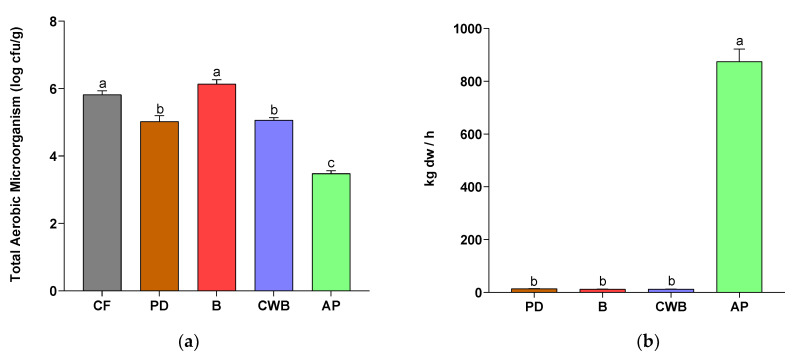
Effect of cleaning treatment on (**a**) total aerobic count and (**b**) profitability of *Agaricus bisporus* stems (ABS) transformation process. Results are reported as mean ± SD (n = 3). Different letters (a–c) in the same graphic are significantly different when subjected to Tukey’s test (*p* < 0.05). CF: control fresh, PD: pre-drying, B: brushing, CWB: immersion in chlorinated water and brushing, AP: abrasive peeling.

Figure 3a shows the total aerobic count in CF, PD, B, CWB, and AP. According to these results, the microbial analyses of the stems under study were influenced by cleaning treatments (*p* < 0.05). There is no difference between CF and B (*p* < 0.05), with aerobic microorganism average counts of 5.82 and 6.14 log cfu/g, respectively, which is on the range analysis of commercialized *A. bisporus* in EU (6-8 log cfu/g) [44,45]. Even when B registered the highest (*p* < 0.05) yield and protein content (Table 1) compared to other cleaning processes, these results showed the inefficiency of the B treatment for washing and preserving food security. Additionally, there is no difference between PD (5.03 log cfu/g) and CWB (5.06 log cfu/g), and both of them were higher than AP (3.48 log cfu/g) (*p* < 0.05). Aerobic microorganisms are indicators of the hygienic quality of products and are also indicative of the potential contamination by pathogenic microbial agents associated with the risk of clinical manifestations [45]. Considering this assertion and the high moisture content of the co-products, which could lead to microbiological growth, AP could be an appropriate cleaning treatment for ABS.

CWB and PD showed, in general, better results in yield, protein content, and total phenolic content than AP (*p* < 0.05) (Table 1). However, hygienic quality is more important than nutritional or functional properties. AP washing treatment resulted in the lowest color degradation (Table 2) and total aerobic count (Figure 3) compared to other cleaning treatments (*p* < 0.05). The lower level of this risk indicator, combined with the subsequent drying process, could enhance the hygienic quality of ABS after AP washing. Nevertheless, the consumption of these food items, including fruiting bodies, in their raw form is discouraged. Since these microorganisms are killed by heat, adequate cooking can protect consumers [45]. Additionally, AP was the most profitable treatment (*p* < 0.05) (Figure 3b) with a production of about 1000 kg per hour, while PD, B, and CWB only gave rise to 12 kg, 14 kg, and 12 kg, respectively. This is because AP is a mechanized method, while the other treatments involve washing the stems individually. Moreover, it is important to note that, being a mechanized process, AP is more reproducible, as it avoids the variability that could occur in industrial-scale processing, where brushing may be performed by different people at varying intensities. 

### 3.2. Drying Process

The comparison of different drying equipment, including a dehydrator, an oven, and a freeze-dryer, is essential for understanding their impact on the quality and appearance of the dehydrated stems. Each drying method involves distinct conditions, such as temperature, drying time, and air exposure, which can influence enzymatic browning and other chemical reactions leading to color changes [43]. Beyond color preservation, the drying process also could degrade bioactive compounds, such as phenolic compounds, which are important due to their antioxidant properties [46]. Additionally, the economic viability of each method must be considered, as cost-effectiveness is crucial for industrial applications. 

#### 3.2.1. Effect of Different Drying Equipment on Color Parameters

The color parameters of the dehydrated stems using different equipment are shown in Table 3. These results show that drying equipment type and species influenced the color parameters (*p* < 0.05). The highest lightness values (L*) and the lowest redness (a*), yellowness (b*), browning index (BI), and chroma (C*) were observed with the freeze-dryer in both species, and also L* and a* with the dehydrator in POS (*p* < 0.05). The highest L* and lowest b* and C* values with FD have been observed in numerous studies in which different drying equipment was compared for drying *A. bisporus* and *P. ostreatus* [42,43,47]. The combined action of freezing and the lower oxygen exposure during the FD process limit the activity of polyphenol oxidase enzymes [48]. Additionally, during the D and HD process, the risen temperature and the oxygen contact may result in enzymatic and non-enzymatic reactions, which lead to a lower L* and higher a*, b*, BI, and C* due to the browning process (*p* < 0.05) [40,47]. 

It is worth mentioning that the color difference (ΔE*) in ABS was higher than in POS with both D and HD (*p* < 0.05). It could be due to the cleaning treatment, which increased surface contact with oxygen due to tissue disruption, resulting in higher color degradation [42]. The L* was lower in HD than in D, as opposed to BI (*p* < 0.05). Additionally, the ΔE* between HD and FD was higher than that of D between FD (*p* < 0.05). These results were consistent with what was observed when drying chicken breast meat and apple fruits with an oven and a dehydrator, the samples dehydrated in a dehydrator showed the highest L*, the least BI and ΔE*, the best visual appearance, and were the most appreciated ones in sensorial analysis [49]. In the oven, the ventilation is constant and more powerful and the environment is more closed, leading to higher exposure of the food to oxygen, promoting polyphenol oxidase activity. As a result, foods dehydrated in an oven tend to experience greater darkening and color degradation compared to those processed in a dehydrator. Color is one of the most important sensory factors in consumer acceptance [49]. According to the results, freeze-drying is the best option for drying the samples. However, this process requires a lot of energy due to the necessity of freezing products, heating the frozen samples to induce sublimation, and lowering the total pressure of the dehydration chamber. As a result, freeze-drying is generally avoided in the food industry [43]. Consequently, dehydration proved to be the most effective method for drying ABS and POS, as it minimized color degradation and energy consumption. Another thing to consider is the involvement of temperature and drying time in enzymatic and non-enzymatic reactions leading to color changes [43,49]. Consequently, it was considered necessary to study the drying curve during the dehydration process at different temperatures.

#### 3.2.2. Effect of Dehydration Temperature on Drying Curves

**Figure 4:**
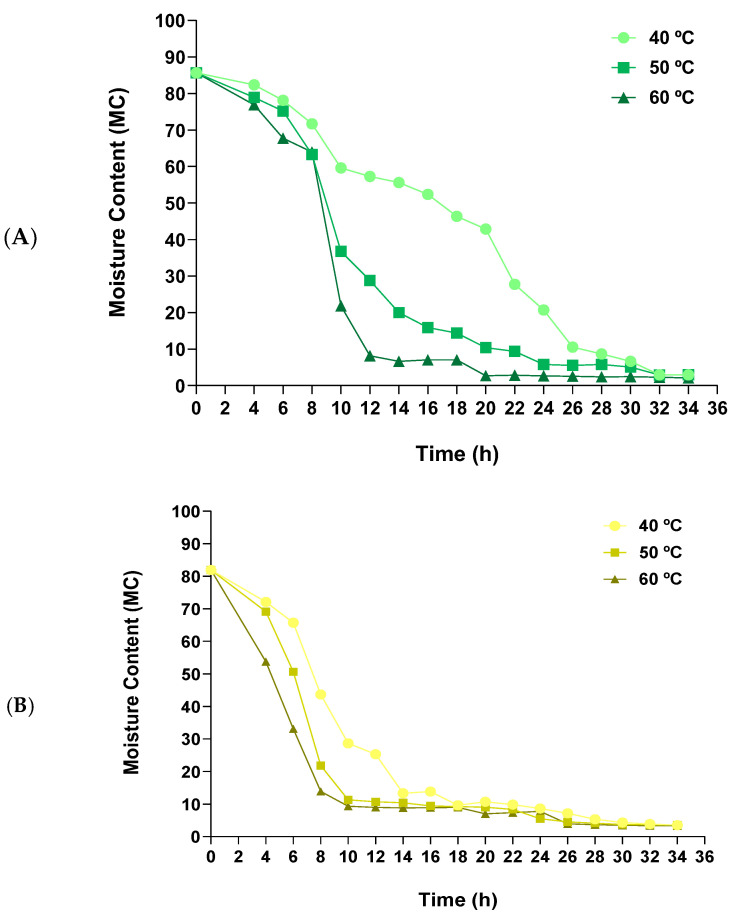
Moisture curve of (**A**) *Agaricus bisporus* stems (ABS) and (**B**) *Pleurotus ostreatus* stems (POS) during the dehydration process at 40, 50, and 60 °C.

Figure 4 shows the moisture content profile during the dehydration of the samples at different temperatures. Higher drying temperatures generally lead to a quicker reduction in moisture content. In ABS (Figure 4A), the effect of temperature on moisture content was more pronounced than in POS (*p* < 0.05). At 40 °C, the moisture content remained above 10% until 28 h of drying, while at 50 °C, this threshold was achieved after 22 h, and at 60 °C it was reached in just 12 h (*p* < 0.05). To attain a moisture content below 5%, 32 h was required at 40 °C, 24 h at 50 °C, and 20 h at 60 °C (*p* < 0.05), indicating a substantial acceleration in the dehydration kinetics with increasing temperature. These results differ significantly from those observed in previous studies, where only 2.7 h at 50 °C was required to achieve a moisture content of 11% and 4.5 h to 6% [50,51]. This may be because, in those studies, sliced fruiting bodies were dried rather than non-sliced stems. For POS (Figure 4B), the moisture content dropped below 10% in 18 h at 40 °C, while at 50 °C and 60 °C, this level was reached at 16 and 10 h, respectively (*p* < 0.05). These results are consistent with those reported by Daud N. et al., where 12 h was required to dry the stems to 10% moisture at 40 and 50 °C, while only 6 h was needed at 60 °C [52]. Furthermore, there was no statistically significant difference (*p* < 0.05) between 50 °C and 60 °C in terms of achieving a moisture content below 5% (24 h), whereas 28 h was necessary at 40 °C. These results suggest that 60 °C may represent the optimal drying temperature, as it substantially reduces the processing time and consequently lowers energy consumption. However, it is necessary to evaluate the potential impact of this higher temperature on the physicochemical properties of the resultant flours, particularly regarding the content of bioactive compounds, which may be sensitive to thermal degradation during the drying process.

#### 3.2.3. Effect of Drying Temperature on Color Parameters

Table 4 shows the evolution of color parameters with the influence of temperature and, consequently, the exposure time. These results show that the studied variables and species generally influenced the color parameters (*p* < 0.05). However, no difference in the browning index (BI) was observed between 40 and 60 °C in POS samples (*p* > 0.05), which may be explained by the longer exposure time at 40 °C, despite the higher temperature at 60 °C. Additionally, at 50 °C, which has the same exposure time as 60 °C (24 h), a lower BI was observed compared to 40 °C (*p* < 0.05), but no difference was found with 60 °C (*p* > 0.05). Browning generally increases with time and temperature through enzymatic and non-enzymatic reactions [49]. The inactivation kinetics of polyphenol oxidase could explain the results of BI in POS. This enzyme in mushrooms experiments a reduction in residual activity by approximately 46% and 92% at 55 °C and 60 °C, respectively, at a treatment time of 30 min [53]. On the contrary, the BI in ABS increased with rising temperature (*p* < 0.05). The tissue disruption by the cleaning treatment in ABS enhanced the interaction between PPO and their substrate [54]. Consequently, the browning index (BI) increased with temperature, even when the residual activity and exposure time were lower [40,47]. The inactivation of PPO and the implication of temperature and time is also evident in the lightness parameter (L*), where no difference is observed between 40, 50, and 60 °C in POS (*p* < 0.05). The lowest L*, the highest yellowness (b*), and chroma (C*) were observed at 60 °C in ABS (*p* < 0.05). Regarding the redness value (a*), it was higher at 60 °C than at 40 °C in both species (*p* < 0.05). Previous works have linked this parameter to non-enzymatic reactions, which are also influenced by temperature [40,47,54]. Therefore, the color degradation observed in a* could be attributed to non-enzymatic reactions in both species. The color difference (ΔE*) in both POS and ABS was higher at 60 °C than at 50 °C due to non-enzymatic reactions in POS and both enzymatic and non-enzymatic reactions in ABS. Therefore, this difference was visible to the human eye at 60 °C in ABS, while in POS, it was invisible at either temperature.

#### 3.2.4. Impact of Drying Temperature on Total Phenolic Content

**Figure 5:**
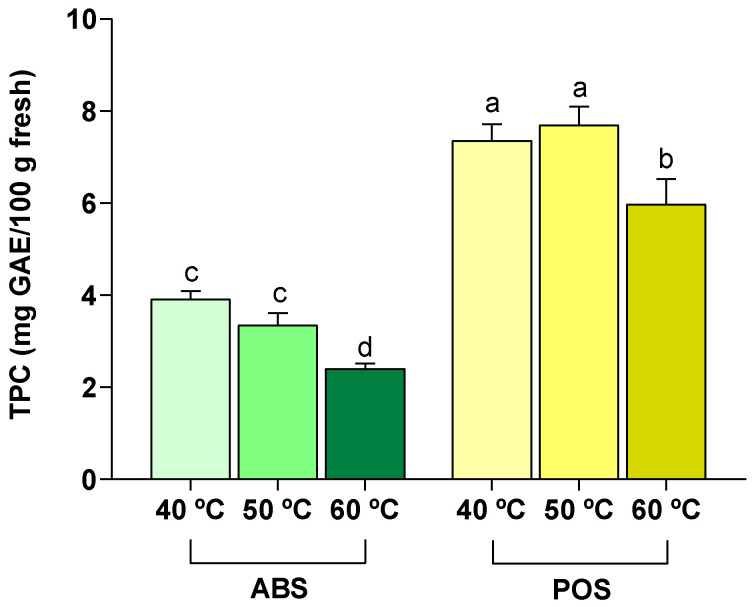
Effect of drying temperature on total phenolic content (TPC) of *Agaricus bisporus* stems (ABS) and *Pleurotus ostreatus* stems (POS). Results are reported as mean ± SD (n = 3). Different letters (a–d) are significantly different when subjected to Tukey’s test (*p* < 0.05).

The values of total phenolic content (TPC) in both ABS and POS are shown in Figure 5. According to these results, drying temperature influenced the TPC evaluated using the Folin–Ciocalteau method (*p* < 0.05). Both species have no difference (*p* < 0.05) between 40 and 50 °C.

The lowest amount observed in each species was 2.40 mg/100 g of ABS fresh and 5.97 mg/100 g of POS fresh, both at 60 °C (*p* < 0.05). These results in ABS agree with what was observed in color parameters concretely in the browning index (Table 4). During cleaning treatment, the tissue could have been damaged, resulting in the consequent release of phenols into the intracellular space, where PPO could have oxidized them [54]. 

It is generally known that the enzymatic and non-enzymatic reactions increased with increasing temperature, and the PPO activity of the mushroom was inactivated at 60 °C [40,47,53]. However, the TPC decreased with increasing temperature even above 60 °C, as shown in previous works, and it could be explained by the samples not reaching the temperature set in the dehydrator or because the initial activity of PPO may have occurred earlier [55]. The highest amount (7.35 and 7.69 mg/100 g of fresh) was obtained for POS at 40 °C and 50 °C, respectively (*p* < 0.05).

This fact contrasts with the obtained results in a previous work, in which *A. bisporus* stems showed higher TPC [56]. However, the results of that work are expressed as g GAE/ g of dry weight, whereas in the present work, they are expressed as g/100 g of fresh, so the difference observed could be due to the yield of ABS after the cleaning treatment (7.29 g/100 g of fresh) compared to the POS yield of 15.01 ± 0.82 g/100 g of fresh. According to the results, 50 °C is the best temperature for drying both species. At this temperature, the drying process of the stems becomes more efficient due to the reduced time required for its ending compared with 40 °C (*p* < 0.05). The shorter processing time not only optimizes agricultural waste revalue, but also decreases the associated economic costs of their transformation. In addition to these benefits, other significant advantages are observed, such as fewer changes in their color parameters and less degradation of bioactive compounds compared to 60 °C.

## 4. Discussion

The results of this study provide valuable insights into the impact of different cleaning and drying treatments on physicochemical characteristics and the profitability of the transformation of ABS and POS into flour. Cleaning treatments were only applied to ABS, with the abrasive peeling (AP) method showing the lowest total aerobic count and the highest profitability. In terms of reproducibility, the mechanized nature of the AP treatment makes it more consistent and less prone to variability, which is an important factor for industrial applications. On the other hand, drying treatments applied to both ABS and POS influenced color parameters and moisture content, with the dehydrator proving to be an efficient method for drying both species. While freeze-drying showed the best preservation of color, the dehydrator offered a good balance between quality preservation and energy efficiency, making it a viable option for large-scale processing. The drying temperature was another important factor influencing both moisture content and bioactive compound preservation. While higher temperatures accelerated the dehydration process, they also caused more substantial degradation of bioactive compounds. Based on the drying curves, 50 °C was identified as the most efficient temperature, balancing faster drying with better retention of TPC and color stability. 

The proposed method, based on the preliminary optimization performed, offers several advantages over composting, particularly in terms of economic value and the nutritional potential of the stems. By converting the stems into flour, a high-value product can be created, which can be used in various food applications, such as bread, pasta, or as a functional ingredient in health supplements [14,25]. This approach preserves and potentially concentrates nutrients like proteins, fibers, and bioactive compounds, contributing to sustainable food systems by reducing waste and providing new, nutrient-rich food sources [56]. On the other hand, while the flour production from the stems provides higher economic returns, it does come with its own set of challenges, as observed in the present project. The processing steps involved—such as cleaning, drying, and milling—require specialized equipment and can be resource-intensive, raising operational costs compared to the simplicity of composting. In addition, obtaining flour from the stems presents other challenges, such as cleaning in the case of ABS, which can be complicated due to the presence of adhering peat. The optimized method (AP) in this project has some limitations, such as water consumption, which may result in an unsustainable process. Future studies could focus on exploring the management of water used in the cleaning process of stems, specifically investigating options like water recirculation and reuse in agriculture. Additionally, the treatment of this water to ensure sustainability and minimize environmental impact should be evaluated. It would also be interesting to continue studying the impact of cleaning treatments on the complete nutritional profile of the stems, as well as other biological activities. Additionally, it would be valuable to investigate the industrial feasibility of the process, as well as the combination of various treatments to ensure both the safety and quality of the final product.

In contrast, composting presents a more straightforward and low-cost solution for disposing agricultural waste. It requires less energy and infrastructure, making it an economically viable option for large-scale agricultural operations. Composting also offers significant benefits by improving soil health and fertility through the addition of nutrient-rich organic matter [57]. However, the composting method results in a low-value product and wastes the nutritional potential of the stems, which could otherwise be utilized in food production. It is also a lengthy process that sometimes cannot meet the requirements of farmers [57]. While composting benefits agriculture by enhancing soil quality, it does not generate a direct consumer market or provide the nutritional and functional benefits that stem flour could. Flour production stands out for its potential to tap into the growing demand for sustainable food ingredients, whereas composting remains a cost-effective option for waste disposal.

## 5. Conclusions

The valorization of agri-food by-products, such as mushroom co-products (ABS and POS), is essential for sustainable development and innovation in the food industry. The chemical composition, physicochemical characteristics, and profitability of transforming these co-products into flour are significantly influenced by cleaning treatments and drying conditions. Cleaning treatments are critical in ensuring the quality and safety of the final product. Preliminary observations suggest that abrasive peeling may reduce aerobic counts and potentially improve profitability compared to other methods, indicating its promise as a cleaning process. This method could offer advantages in enhancing the hygienic quality and reproducibility of ABS flour production, but further research is needed to fully assess its effectiveness and scalability. A pilot-scale study would be necessary to assess the techno-economic and environmental impacts of this process. Additionally, exploring the combination of various methods, such as using chlorinated water during the AP cleaning process, could further optimize the results. Regarding the drying process, dehydration in a dehydrator at 50 °C for 24 h seemed to provide a favorable balance between cost-efficiency and minimal degradation of phenolic compounds and color changes in both ABS and POS. Transforming these co-products into stable forms, such as flour, could extend their shelf life, potentially ensuring longer-term quality and safety. While further optimization and more extensive studies are required, our results suggest that improved cleaning and drying processes could enhance the quality of these co-products for use in various food industry applications. Ultimately, this approach has the potential to contribute to waste reduction, and it also supports a circular economy model, offering a sustainable direction for resource utilization in the food industry.

## Figures and Tables

**Table 1:** Effect of different cleaning treatments on MC, yield, protein content, and TPC of *Agaricus bisporus* stems (ABS).

	MC	Yield	Protein Content	TPC
CF	81.00 ± 0.67 ^c^	18.69 ± 0.67 ^a^	0.96 ± 0.03 ^a^	13.30 ± 0.38 ^b^
PD	81.00 ± 0.99 ^c^	12.05 ± 0.52 ^c^	0.39 ± 0.01 ^c^	12.54 ± 0.66 ^c^
B	85.00 ± 0.26 ^b^	14.16 ± 0.52 ^b^	0.65 ± 0.03 ^b^	12.04 ± 0.56 ^c^
CWB	88.00 ± 0.46 ^a^	12.14 ± 0.07 ^c^	0.42 ± 0.03 ^c^	15.99 ± 0.66 ^a^
AP	87.00 ± 0.23 ^a^	7.29 ± 0.39 ^d^	0.26 ± 0.00 ^d^	5.36 ± 0.16 ^d^

Results are reported as mean ± SD (n = 3). Mean values within columns followed by different superscript letters (a–d) are significantly different when subjected to Tukey’s test (*p* < 0.05). CF: Control fresh, PD: Pre-drying, B: Brushing, CWB: Immersion in Chlorinated Water and Brushing, AP: Abrasive Peeling, MC: Moisture Content, TPC: total phenolic content. Results are expressed as g/100 g of fresh, TPC as mg GAE/100 g fresh.

**Table 2:** Effect of different cleaning treatments on color parameters of *Agaricus bisporus* stems (ABS).

	L*	a*	b*	BI	C*	ΔE*
CF	67.82 ± 1.19 ^d^	2.31 ± 0.14 ^e^	9.37 ± 0.18 ^e^	16.86 ± 0.68 ^e^	9.65 ± 0.20 ^e^	-
PD	68.19 ± 0.47 ^c,d^	5.38 ± 0.10 ^a^	17.22 ± 0.10 ^a^	34.01 ± 0.41 ^a^	18.04 ± 0.11 ^a^	24.14 ± 0.78 ^a^
B	70.27 ± 1.12 ^b,c^	4.97 ± 0.09 ^b^	16.51 ± 0.17 ^b^	31.15 ± 0.73 ^b^	17.24 ± 0.19 ^b^	19.20 ± 1.06 ^b^
CWB	71.56 ± 0.61 ^a,b^	4.42 ± 0.08 ^c^	14.73 ± 0.17 ^c^	26.82 ± 0.44 ^c^	15.37 ± 0.18 ^c^	11.94 ± 0.69 ^c^
AP	73.22 ± 0.64 ^a^	3.90 ± 0.07 ^d^	14.08 ± 0.09 ^d^	24.57 ± 0.19 ^d^	14.61 ± 0.08 ^d^	9.18 ± 0.27 ^d^

Results are reported as mean ± SD (n = 3). Mean values within columns followed by different superscript letters (a–e) are significantly different when subjected to Tukey’s test (*p* < 0.05). CF: control fresh, PD: pre-drying, B: brushing, CWB: immersion in chlorinated water and brushing, AP: abrasive peeling, L*: lightness, a*: redness, b*: yellowness, C*: chroma, BI: browning index, ∆E*: color difference.

**Table 3:** Effect of drying equipment type on color parameters of *Agaricus bisporus* stems (ABS) and *Pleurotus ostreatus* stems (POS).

	Equipment	L*	a*	b*	BI	C*	ΔE*
ABS	FD	84.10 ± 2.00 ^a^	1.67 ± 0.39 ^c,d^	8.27 ± 0.58 ^d^	11.46 ± 1.33 ^e^	8.44 ± 0.64 ^d^	-
	D—40 °C	73.22 ± 0.64 ^c^	3.90 ± 0.07 ^b^	14.08 ± 0.09 ^c^	24.57 ± 0.19 ^c^	14.61 ± 0.08 ^c^	16.91 ± 0.57 ^b^
	HD—40 °C	64.89 ± 0.62 ^d^	4.67 ± 0.18 ^a^	14.60 ± 0.26 ^c^	29.99 ± 0.91 ^b^	15.33 ± 0.30 ^c^	28.18 ± 0.63 ^a^
	FD	84.03 ± 0.26 ^a^	0.98 ± 0.09 ^e^	16.53 ± 0.23 ^b^	22.03 ± 0.46 ^d^	16.56 ± 0.23 ^b^	-
POS	D—40 °C	81.62 ± 0.58 ^a^	1.43 ± 0.13 ^d,e^	20.95 ± 0.35 ^a^	29.95 ± 0.92 ^b^	21.00 ± 0.36 ^a^	2.75 ± 0.17 ^d^
	HD—40 °C	78.36 ± 0.84 ^b^	2.10 ± 0.11 ^c^	21.15 ± 0.19 ^a^	32.38 ± 0.63 ^a^	21.00 ± 0.20 ^a^	7.38 ± 0.42 ^c^

Results are reported as mean ± SD (n = 3). Mean values within columns followed by different superscript letters (a–e) are significantly different when subjected to Tukey’s test (*p* < 0.05). FD: freeze-dryer, D: dehydrator, HD: hot-air dryer, L*: lightness, a*: redness, b*: yellowness, C*: chroma, BI: browning index, ∆E*: color difference.

**Table 4:** Effect of drying temperature on color parameters of *Agaricus bisporus* stems (ABS) and *Pleurotus ostreatus* stems (POS).

	°C	L*	a*	b*	BI	C*	ΔE
ABS	40–32 h	73.04 ± 0.42 ^b^	4.29 ± 0.14 ^b^	14.91 ± 0.31 ^d^	26.42 ± 0.82 ^e^	15.52 ± 0.33 ^e^	-
	50–24 h	70.95 ± 0.69 ^b^	4.77 ± 0.05 ^a,b^	15.92 ± 0.23 ^d^	29.56 ± 0.61 ^d^	16.62 ± 0.23 ^d^	2.43 ± 0.33 ^b^
	60–20 h	66.81 ± 0.90 ^c^	5.38 ± 0.08 ^a^	17.66 ± 0.19 ^c^	35.69 ± 0.54 ^a^	18.47 ± 0.19 ^c^	6.94 ± 0.81 ^a^
	40–28 h	84.51 ± 1.43 ^a^	1.81 ± 0.50 ^d^	23.56 ± 0.38 ^a^	33.16 ± 0.88 ^b^	23.64 ± 0.36 ^a^	-
POS	50–24 h	84.79 ± 0.37 ^a^	1.87 ± 0.16 ^d^	22.13 ± 0.63 ^b^	30.86 ± 1.19 ^c,d^	22.21 ± 0.64 ^b^	0.40 ± 0.06 ^c^
	60–24 h	83.68 ± 0.47 ^a^	2.58 ± 0.17 ^c^	22.09 ± 0.34 ^b^	31.90 ± 0.77 ^b,c^	22.24 ± 0.35 ^b^	1.65 ± 0.21 ^b^

Results are reported as mean ± SD (n = 3). Mean values within columns followed by different superscript letters (a–e) are significantly different when subjected to Tukey’s test (*p* < 0.05). L*: lightness, a*: redness, b*: yellowness, C*: chroma, BI: browning index, ∆E*: color difference.

## Data Availability

The original contributions presented in the study are included in the article, further inquiries can be directed to the corresponding author.
